# Membrane and Cytoplasmic Proteins of *Mycobacterium avium* subspecies *paratuberculosis* that Bind to Novel Monoclonal Antibodies

**DOI:** 10.3390/microorganisms6040127

**Published:** 2018-12-11

**Authors:** John P. Bannantine, Judith R. Stabel, John D. Lippolis, Timothy A. Reinhardt

**Affiliations:** 1 Infectious Bacterial Diseases Research Unit, National Animal Disease Center, USDA-Agricultural Research Service, Ames, IA 50010, USA; judy.stabel@ars.usda.gov; 2 Ruminant Diseases Immunology Research Unit, National Animal Disease Center, USDA-Agricultural Research Service, Ames, IA 50010, USA; john.lippolis@ars.usda.gov (J.D.L.); tim.reinhardt@ars.usda.gov (T.A.R.)

**Keywords:** Johne’s disease, *Mycobacterium avium* subspecies *paratuberculosis*, monoclonal antibodies, proteomics

## Abstract

Monoclonal antibodies against *Mycobacterium avium* subspecies *paratuberculosis* *(Map)* proteins are important tools in Johne’s disease research and diagnostics. Johne’s disease is a chronic inflammatory intestinal disease of cattle, sheep, and other ruminant animals. We have previously generated multiple sets of monoclonal antibodies (mAbs) in different studies; however, because many were generated and screened against a whole-cell extract of *Map*, the antigens that bind to these antibodies remained unknown. In this study, we used three different approaches to identify the corresponding *Map* antigens for 14 mAbs that could not be identified previously. In the first approach, a new *Map*-lambda phage expression library was screened to identify corresponding antigens for 11 mAbs. This approach revealed that mAbs 7C8, 9H3, 12E4, 3G5, and 11B8 all detect MAP_3404 encoding the biotin carboxylase subunit of acetyl-CoA carboxylase, while mAbs 7A6, 11F8, and 10C12 detect the GroEL2 chaperonin (MAP_3936), 6C9 detects electron transfer flavoprotein (MAP_3060c), and 14G11 detects MAP_3976, a lipoprotein anchoring transpeptidase. The epitopes to a selection of these mAbs were also defined. In a second approach, MAP_2698c bound monoclonal antibody (mAb) 14D4 as determined using protein arrays. When both of these approaches failed to identify the antigen for mAb 12C9, immunoprecipitation, mass spectrometry analysis, and codon optimization was used to identify the membrane protein, MAP_4145, as the reacting antigen. Characterized antibodies were used to quickly interrogate mycobacterial proteomic preps. We conclude by providing a complete catalog of available mAbs to *Map* proteins, along with their cognate antigens and epitopes, if known. These antibodies are now thoroughly characterized and more useful for research and diagnostic purposes.

## 1. Introduction

Monoclonal antibodies (mAbs) are important detection reagents for diagnostics and therapeutic purposes. One exciting and recent application of mAb technology to combat bacterial infection showed that mice colonized with *Klebsiella* in the gut and treated with mAbs can be protected against antibiotic-induced dissemination from the gut [1]. This suggests potential new therapeutic strategies for the systemic treatment with monoclonal antibodies against gut-colonizing bacteria, of which *Mycobacterium avium* subspecies *paratuberculosis* (*Map*) is a member. However, very few monoclonal antibodies have been produced against *Map* and even less have been characterized. For example, two monoclonal antibodies have recently been used to capture *Map* when coated onto magnetic beads; however, the *Map* protein these antibodies bind to remains unknown [2]. The same antigen anonymity is true for mAbs that react to secreted proteins of *Map* [3].

To close a research gap and facilitate detection of *Map*, which causes a chronic intestinal disease in cattle, sheep, and goats, we previously generated a set of stable hybridomas against *Map* whole cell extracts or membrane enriched extracts [4,5]. In those studies, only five antigens were successfully identified when screening a phage lambda expression library with the antibodies. The DnaK chaperone (MAP_3840) was identified as the corresponding antigen for mAbs 11G4 and 13A4, along with isocitrate lyase enzyme (MAP_1643) for mAbs 9G10 and 11F6 [4]. A proline-rich antigen (MAP_1025) was later identified using a similar screening approach with mAb 17A12 [5]. However, the remaining five mAbs failed to react with plaques in the phage library and thus their cognate antigens remained unknown. One of these antibodies (4B6) detected a highly conserved protein among all tested mycobacterial species [4]. Because of this lack of specificity, mAb 4B6 was not pursued further. A subsequent study generated 22 additional mAbs in our laboratory that were not published and corresponding antigens were never identified. Those mAbs were examined further in this study.

In separate studies, our group also obtained mAbs to select proteins of interest using well-defined recombinant proteins as the immunizing antigen in mice. For example, two mAbs were obtained when immunizing mice with MAP_1272c, a strong antigen that has been shown to hydrolyze peptidoglycan [6,7]. This antigen is a NlpC/P60 domain containing protein that was recently crystalized and shown to have lost the ability to cleave peptidoglycan due to a single amino acid modification in the catalytic triad [7]. The two mAbs successfully developed to this protein each bound distinct epitopes in MAP_1272c [6]. Another two mAbs were obtained when immunizing mice with the 35-kDa major membrane protein [8]. In each study, there was no need to define the cognate antigen, because the recombinant proteins used for immunizing mice were well characterized [7,9]. However, in several other attempts, mAbs were not successfully obtained with recombinant proteins, or more commonly, the resulting hybridoma secreting antibodies only reacted to the *E. coli* expressed recombinant and not the native *Map* protein, highlighting the limitation of this approach.

Although we had success identifying cognate antigens by screening a phage expression library of *Map* K-10 for several monoclonal antibodies, there were still a number of antibodies that did not show reactivity using this type of screening method. This result was reproducible even after three independent attempts at different times with different personnel. Use of antibodies that bind to unknown antigens in studies can lead to error-prone conclusions. For example, a PD4 mAb was used in cancer research because it specifically bound to tumor cells. The antibody was obtained by immunizing mice with the human gastric cell line MGC803 [10]. However, it was later discovered after a failed cDNA expression library screen that the antigen to PD4 was a membrane protein of *Mycoplasma hyorhinis* that could bind directly to tumor cells [11]. Therefore, when library screening approaches failed, we pursued immunoprecipitation and protein array approaches to identify remaining antigens.

We conclude this study by using the newly acquired information to determine relative abundance of selected proteins among the *Mycobacterium avium* complex (MAC) as an example of how these reagents can quickly interrogate the quality of proteomic preparations. This catalog of monoclonal antibodies should prove useful for *Map* research.

## 2. Materials and Methods

### 2.1. Map Culture and Whole Cell Extract Preparation

*Map* bovine strain K-10 and ovine strain S397 were cultured in Middlebrooks 7H9 medium (Becton Dickinson, Franklin Lakes, NJ, USA) and supplemented with 2 mg of mycobactin J (Allied Monitor Inc., Fayette, MO, USA) per liter and 10% oleic acid-albumin-dextrose complex (OADC; Becton Dickinson) plus 0.05% Tween 80 (Sigma-Aldrich, St. Louis, MO, USA) as described previously [12]. The S397 strain was used in construction of the genomic expression library and the K-10 strain was used for whole cell antigen in this study. Antigen preparations were harvested by centrifugation, washed in phosphate buffered saline (PBS; 10 mM PO_4_, 137 mM NaCl, and 2.7 mM KCl, pH 7.4.), and sonic disrupted as described by Waters et al. [12].

### 2.2. Map-Lambda ZAP Expression Library Construction and Screening

The *Map* 397 expression library was prepared and used as previously described for the *Map* ATCC19698 library [13]. Briefly, DNA from the ovine S397 strain of *Map* was restriction digested using *Sau*3AI (New England Biolabs, Beverly, MA, USA) and fragments in the 4 to 6 kilobase size range were selected for cloning into *Bam*HI predigested lambda ZAP arms (Stratagene, La Jolla, CA, USA). *E. coli* XL1-Blue MRF’ was used as the host strain for infection with the lambda ZAP phage vector. Pilot and scale up platings were conducted on NZY agar plates (3% [wt·vol^−1^] N-Z Amine A (Sigma-Aldrich), 1% [wt·vol^−1^] yeast extract [Fisher Scientific], pH 7.5), and supplemented as necessary with either 1.5% or 0.7% (wt·vol^−1^) agarose (Lonza, Allendale, NJ, USA). Each plate also contained Isopropyl-Beta-d-Thiogalactoside (IPTG; 0.01 M) and 5-bromo-4-chloro-3-indolyl-β-d-galactopyranoside (X-gal; Sigma-Aldrich), which demonstrated greater than 85% of the plaques contained inserts. Nitrocellulose plaque lifts were created from each plate and exposed to mAbs that were mixed equally (10 μg/mL) and diluted 1:200 in a blocking buffer consisting of PBS with 2% bovine serum albumin (BSA) and 0.1% tween 20 (Sigma). Plaques reacting with mAbs were subcloned by infection of *E. coli* XLOLR strain (Stratagene) with the phage lysate obtained from the positive plaque and the addition of ExAssist helper phage (Stratagene). This infection resulted in the in-vivo excision of pBK-CMV phagemid containing *Map* genes of interest. The excision subclones were selected on NZY agar (1.0% NZ amine, 0.5% NaCl, 0.5% yeast extract, 1.5% agar) with kanamycin at 50 µg/mL and were numbered consecutively (i.e., phage clone #1, #2, #3, etc).

### 2.3. Cloning and Expression of Selected Map Open Reading Frames

Most of the recombinant proteins in this study were obtained from an established collection of *Map* ORF expression clones in our laboratory [14]. MAP_4145 was not among this collection, but attempts to amplify the GC-rich coding sequence from strain K-10 using either dimethyl sulfoxide (Sigma-Aldrich), GC advantage-2 (Clonetech, Mountainview, CA, USA), or Econo-Taq Plus GREEN PCR master mix (Lucigen, Middleton, WI, USA) for cloning and expression all failed. However, the gene was ultimately codon optimized for *E. coli* and chemically synthesized as dsDNA gBlocks (Integrated DNA Technologies, Coralville, IA, USA) with 30 base-pair complimentary terminal sequences to the linearized *Nde*I-*Xho*I (FastDigest; Thermo Scientific, Minneapolis, MN, USA) pET30a vector for cloning via the isothermal assembly method [15]. To each isothermal assembly reaction, 25 femtomoles of each gBlock was added to 75 femtomoles of linearized vector and incubated at 50 °C for 1 h. One tenth of each reaction was transformed into chemically competent *E. coli* DH5-α host cells and recombinant clones selected for on LB solid agar medium (BD Difco, Franklin Lakes, NJ, USA), supplemented with 50 μg/mL kanamycin (Sigma-Aldrich). All cloned inserts were confirmed by DNA sequencing. Confirmed clones were expressed and recombinant proteins were purified as previously described [16]. Cloned inserts were aligned to the full-length proteins using MacVector version 16.0.8 (MacVector, Raleigh, NC, USA).

### 2.4. Immunoblot and Dot Blot Analysis

Sodium dodecyl sulfate–polyacrylamide gel electrophoresis (SDS-PAGE) and immunoblot analysis were conducted using standard methods as previously described [12]. Protein size standards used in this study were from two commercial suppliers (Thermo Fisher and Bio-Rad). The dot blot assay was performed using a Bio-Dot 96-well manifold assembly (Bio-Rad, Hercules, CA, USA) following manufacturer instructions. A range from 0.5 to 1 g of each protein was spotted onto nitrocellulose sheets using methods previously described [17]. The monoclonal antibodies were diluted anywhere from 1:50 to 1:1000, depending on high or low secreting hybridomas that produce the mAb.

The dot blot of full-length proteins was constructed from available recombinant proteins in our laboratory [14]. Pools of 2, 3, or 4 recombinant proteins were combined in each spot with spot assignments shown in supplemental Table S1. Dot blots of overlapping peptides representing specific proteins in this study were treated similarly except that distilled water, instead of PBS, was used as the diluent. The anti-mouse-HRP (Thermo Scientific) was diluted 1:20,000. Nitrocellulose blots were developed with Super signal (Thermo Fisher) and exposed Biomax MR film (Carestream Health, Inc., Rochester, NY, USA). Exposed film was processed using a PROTEC EcoMAX X-ray film processor (Oberstenfeld, Germany). For the quantitative dot blot experiments, mycobacterial sonicated protein extracts were initially quantitated using the Lowery protein assay (Thermo Scientific). Equivalent amounts of extract from each strain were diluted and loaded onto nitrocellulose sheets using the Bio-Dot 96-well manifold assembly.

### 2.5. ELISA Assay

A homemade ELISA was conducted for mycobacterial reactivity studies and epitope mapping studies. Briefly, peptides were all reconstituted at 1.0 to 1.5 mg/mL concentrations in sterile DNase-free distilled water and stored, as described in Section 2.7. Nunc polysorb 96-well plates were coated with 1 μg of each peptide or mycobacterial extract per well in coating buffer (0.05 M carbonate-bicarbonate buffer, pH 9.6) overnight at 4 °C. Sequence and location for the peptide arrays are listed in the supplemental Table S2. Plates were washed three times in PBS-0.05% tween 20 and blocked at room temperature for 1 h in 1% BSA-PBS-0.05%, tween 20. Next, three washes were performed as above and monoclonal antibodies were diluted in 1% BSA-PBS-0.05% tween 20 and added to the plate (100 µL/well) for 1 h at room temperature. Three washes were again performed as above and anti-mouse-HRP (Thermo Fisher) was diluted 1:20,000, added to each well and incubated for 1 h. A final three washes were performed and SureBlue TMB-1 substrate (KPL, Gaithersburg, MD, USA) was added for 5–10 min. The reaction was stopped in TMB stop solution (KPL) and plates were read at 450 nm in a SpectrMax340 spectrometer (Molecular Devices, San Jose, CA, USA).

### 2.6. Antigen Capture and Mass Spectroscopy

The *Map* protein that binds to mAb 12C9 was captured by an indirect and direct immunoprecipitation. The *Map* K-10 extract was biochemically fractionated and each fraction was examined for the presence of the antigen. The fraction with the strongest reactivity to 12C9 was used in the immunoprecipitation experiment. To isolate the 12C9-antigen complex via the indirect method, 500 µg of extract was mixed with 0.8 µg 12C9 antibody in 1× PBS buffer for 1 h followed by addition of magnetic sheep anti-mouse IgG Dynabeads (Novex/Life Technologies) for 30 min at room temperature. To induce release of antigen from the 12C9-bead complex, the beads were washed 2 times in 1× PBS and then boiled in 2× SDS loading buffer for 5 min and subjected to SDS-PAGE and GelCode Blue staining. Sections were excised from the polyacrylamide and processed for mass spectrometry. The direct capture method was similar to above except that the antibody was exposed to the Dynabeads for 30 min prior to a 1-h incubation with the extract.

The excised gel slices were trypsin digested, dried, and analyzed on a nano-LC chromatography using a Proxeon Easy-nLC connected to LTQ OrbiTrap Velos Pro mass spectrometer (ThermoFisher Scientific, West Palm Beach, FL, USA) as described previously [18].

### 2.7. Epitope Mapping Studies

For selected proteins, an approximate epitope was determined by cloning and expressing segments of the protein, in other cases, an overlapping peptide library was synthesized and used to determine the epitope location. Peptide libraries were constructed (Thermo Scientific) for 2 *Map* proteins, MAP_2698c, and MAP_3404. The peptide lengths, sequences, location within each array, and degree of amino acid overlap are indicated in supplemental Table S2. The design strategies resulted in 86 total peptides for the MAP_3404 array and 52 total peptides for the MAP_2698c array. Each array also included the full length maltose binding protein (MBP) fusion protein as a positive control. Each peptide was dissolved in distilled water to yield a concentration of between 1 and 1.5 mg/mL and stored in ultra-low attachment polystyrene 96-well plates (Corning, Kennebunk, ME, USA) at −20 °C with a working stock stored at 4 °C. Peptides that were less soluble were gently heated (45 °C for 10 min) to hasten dissolving. For crude epitope mapping experiments, cloning and expression of truncated MBP fusion proteins was performed as described previously [14].

Peptides used for dot blot arrays were spotted onto nitrocellulose sheets using the Bio-dot apparatus as described above for constructing the protein arrays. These nitrocellulose peptide arrays were blocked and processed in the same manner as described for immunoblots. For ELISA assays, peptides were coated onto ELISA plates and processed as described in Section 2.5.

## 3. Results

### 3.1. Monoclonal Antibodies to Map Proteins

Collectively, our laboratory has produced a total of 36 mAbs against *Map*. Of these, ten were previously produced using a whole cell extract of *Map* as the immunizing antigen [4]. Another 22 mAbs were recently generated against *Map* extracts and characterized in this study. And finally, four mAbs were generated by immunizing mice with recombinant proteins to MAP_1272 [6] and MAP_2121c [8]. All 36 antibodies were characterized by their reactivity with a panel of 15 mycobacteria representing the *M. avium* and *M. tuberculosis* complexes, as well as fast growing mycobacteria (supplemental Table S3). Only one mAb was specific for the *paratuberculosis* subspecies of *M. avium* (17A12), which has also been confirmed previously [5]. In contrast, mAb 4B6 binds to a highly conserved protein present in all mycobacterial species tested (Table S3). The rest of the antibodies showed varying degrees of cross-reactivity that resembled genetic similarity across the mycobacteria. For example, 88–100% of all antibodies reacted to all three *Map* strains tested, whereas only 6% of all antibodies reacted to the more distantly related *M. smegmatis* (Table S3).

The cognate antigens for most of these mAbs is unknown despite previous work screening a *Map* ATCC19698-lambda expression library [4]. The mAbs in that study could not be identified from the same expression library despite repeated efforts. Therefore, different approaches were used to identify the corresponding antigens for several mAbs.

### 3.2. Monoclonal Antibody Screening Using the Sheep S397 Expression Library

**Figure 1:**
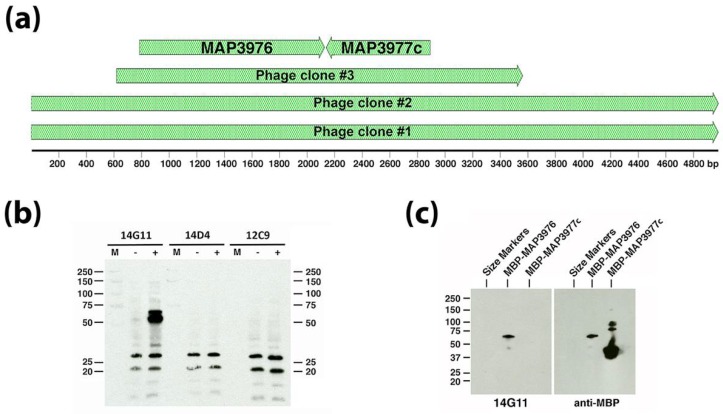
Antibody 14G11 reacts with MAP_3976. A genomic expression library screen identified three phage plaques that reacted to a cocktail of 3 monoclonal antibodies (mAbs). Sequence alignments for the lambda clone inserts are shown to scale in (**a**) along with coding sequences from the *Mycobacterium avium* subspecies *paratuberculosis* (*Map)* K-10 genome present within the smallest insert of phage clone #3. The scale is shown in base pairs. Phage clone #3 was analyzed by immunoblot under IPTG inducing and non-inducing conditions as indicated by a “+” or “−“ above each immunoblot in (**b**) and three identical immunoblots were exposed to the three mAbs used in the original library screen and indicated above each blot. Protein size markers (M) are indicated in the left and right margins in kilodaltons. Only mAb 14G11 reacted to an IPTG induced 50-kDa protein expressed from clone #3 (left blot in (**b**)). (**c**) MAP_3976 and MAP_3977c were cloned and expressed as maltose binding protein (MBP) fusions and immunoblot analysis confirmed that only MAP_3976 reacted to 14G11 (left blot) while both fusion proteins reacted to the MBP tag (right blot).

Because 12C9, 14D4, and 14G11 did not yield positive plaques from screening a *Map* bovine strain (ATCC19698) expression library [4], a second genomic DNA expression library was cloned from the *Map* ovine isolate S397 [19] and screened with the three combined mAbs. Obtained from this screen were three positive reacting plaques and the inserts from these plaques were subcloned and sequenced (Figure 1A). Two of the phage clones had inserts containing the same start and stop coordinates and was 4980 bp in length while phage clone #3 had beginning and ending coordinates within the first two phage clones and totaled 2943 bp (Figure 1A). All clones overlapped with a region encompassing two coding sequences (MAP_3976 and MAP_3977c) in the K-10 genome. When the phage clone #3 insert was subcloned into *E. coli* and induced to express the insert, only mAb 14G11 reacted with a protein around 50 kDa in size (Figure 1B). The calculated molecular weight for MAP_3976 is 47 kilodaltons (kDa), while MAP_3977c is only 26 kDa. Furthermore, the direction of transcription of the lacZ promoter within the lambda phage clones is indicated by the arrows in Figure 1A and is in frame with MAP_3976 but on the opposite strand for MAP_3977c. These observations combined to suggest that the MAP_3976 expression product reacts with mAb 14G11. To confirm this, both genes were cloned and expressed in *E. coli* separately. Only MAP_3976 reacted with 14G11 when the purified recombinant proteins were analyzed by immunoblot analysis (Figure 1C). This protein is a lipoprotein anchoring transpeptidase.

A set of 22 additional mAbs were screened using the original ATCC19698 and the newly constructed S397 phage libraries. Among these antibodies, 12 reacted with at least 1 plaque from either library. Three antibodies (1C8, 8C6, and 10D2) bound to the isocitrate lyase protein (MAP_1643), adding to two mAbs (9G10 and 11F6) that had been obtained in a previous study [4]. Five antibodies bound to acetyl-CoA carboxylase (MAP_3404), three mAbs bound to the chaperone GroEL2 (MAP_3936), and one mAb reacted to an electron transfer protein (MAP_3060c) (supplemental Figures S1 and S2). However, no positive lambda plaques were obtained that had reactivity with two mAbs, 12C9 and 14D4, from our initial study [4]. Therefore, different approaches were needed to identify their respective binding antigens.

### 3.3. Dot Blot Array Identifies the Cognate Antigen of mAb 14D4

**Figure 2:**
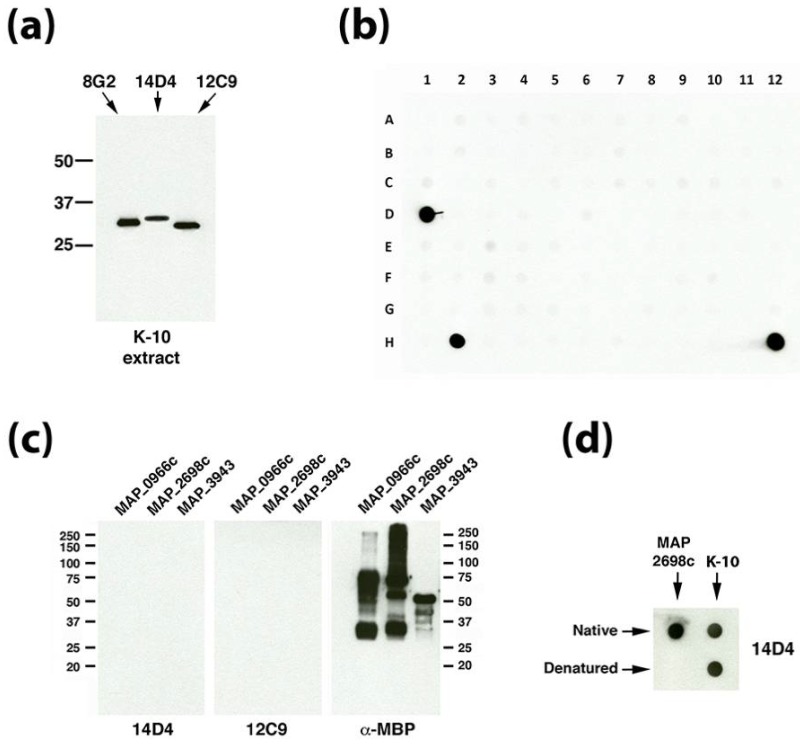
MAP_2698c reacts with mAb 14D4 through a conformation epitope. (**a**) Immunoblot analysis with mAbs 14D4 and 12C9 on the whole cell extract of *Map* K-10 shows the similar molecular sizes of each protein. These are compared to the 8G2 mAb that binds to the major membrane protein (MAP_2121c, [8]), which has a calculated molecular weight of 33.6 kDa. (**b**) Dot blot array containing 355 spotted recombinant proteins (see supplemental Table S1 for spot assignments) was exposed to 12C9 and 14D4. While spots D1 and H12 contain positive controls, spot H2 contains a mixture of recombinant proteins MAP_0966c, MAP_2698c and MAP_3943. (**c**) Three identical immunoblots of each recombinant protein are exposed to mAbs indicated beneath each blot. MAbs 14D4 and 12C9 show no reactivity to any proteins while the α-MBP control, which binds to the affinity tag, shows proteins are heavily loaded and immunoreactive. (**d**) Dot blot analysis demonstrates that 14D4 reacts with the recombinant MAP_2698c, but only under native conditions. The native protein from the K-10 extract is not subject to this limitation. Kilodalton molecular size markers are indicated in (**a**,**c**).

One characteristic that links the unknown antigens that bind mAbs 12C9 and 14D4 is their similar molecular size based on migration in SDS-PAGE gels. Their estimated size is 30 to 35 kDa based on similarity to the known 33.5-kDa major membrane protein (Figure 2a) and comparison with two commercially available protein marker sets. This size criterion was used to screen recombinant *Map* proteins available from our collection [14]. There are 1,988 annotated proteins with calculated molecular sizes between 20–41 kDa [20]. Our collection had 355 purified *Map* recombinant proteins available within this size range, which represents 18% of all *Map* proteins in that range. These recombinant proteins were arranged in a dot blot protein array format and exposed to mAbs 14D4 and 12C9 (Figure 2b). Data show that the mAb mixture reacted strongly to three spots on the array, two of which included the whole cell extract of strain K-10 which served as the positive control (spots D1 and H12 in Figure 2b). The recombinant proteins present in spot H2 included MAP_0966c, MAP_2698c, and MAP_3943. When the proteins and mAbs were separated out and analyzed individually by denaturing immunoblot, reactivity disappeared (Figure 2c). Subsequent experiments demonstrated that MAP_2698c is detected by the 14D4 antibody and that only the native form of the protein is recognized by the antibody (Figure 2d). To determine if the epitope could be located, a MAP_2698c peptide array was constructed (supplemental Table S2). However, mAb 14D4 appears to bind a conformational epitope since it did not bind to the MAP_2698c peptide array (data not shown) and does not react in denaturing Western blot assays (Figure 2c). MAP_2698c is an acyl carrier protein desaturase that may be involved in mycolic acid biosynthesis and has recently been shown to be antigenic and located in the cell envelop [21].

### 3.4. Identification of the 12C9 Binding Antigen by Mass Spectrometry

**Figure 3:**
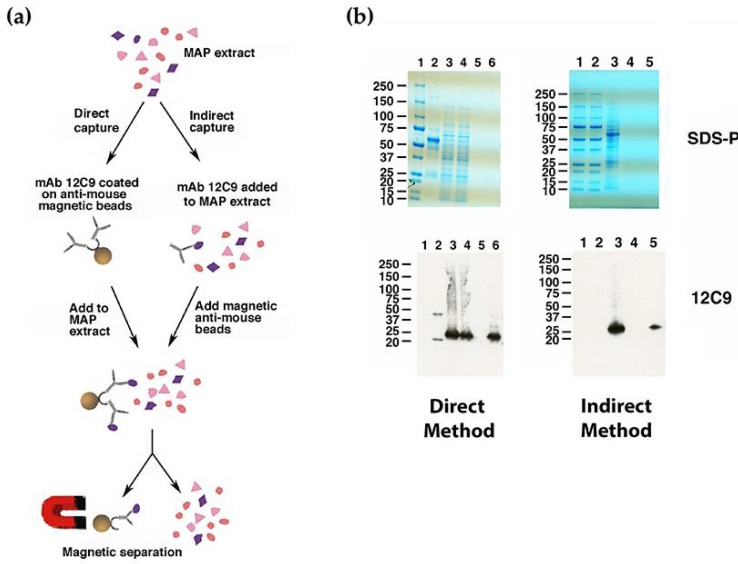
Direct and indirect capture of the *Map* antigen that binds mAb 12C9. Shown is a schematic flow diagram of the two methods (**a**). The primary difference between these methods is that the mAb is bound to the magnetic beads before exposure to the antigen extract versus afterwards as in the indirect method. The brown sphere is the magnetic bead. Both methods were analyzed by SDS-PAGE and immunoblot analysis (**b**). Shown are stained sodium dodecyl sulfate–polyacrylamide gel electrophoresis (SDS-PAGE) gels (top) and corresponding immunoblots probed with 12C9 (bottom) for the direct (left) and indirect (right) capture experiments. The 12C9 probed blots show that the antigen was captured while the anti-mouse control had no reactivity suggesting that the capture was specific. Lanes for the direct method are: 1 = Protein size markers, 2 = bead coupled 12C9 antibody, 3 = K-10 antigen before capture, 4 = K-10 antigen after capture with 12C9 magnetic beads, 5 = combined washes, and 6 = antigen eluted from magnetic beads. Lanes for the indirect method are: 1 and 2 = Protein size standards, 3 = K-10 antigen mixed with 12C9 antibody, 4 = combined washes after incubation, and 5 = antigen eluted from magnetic beads.

The 12C9 mAb could not be identified by either a phage library screen or using the dot blot array of size-selected proteins, yet hybridoma cells strongly secrete this antibody and immunoblot analysis suggests it binds to a protein approximately 30 kDa (Figure 2a) with strong affinity. Therefore, the 12C9 antibody was used to capture and enrich for its binding partner via immunoprecipitation with magnetic beads. Both a direct and indirect capture was conducted (Figure 3a); however, the direct method captured more antigen (Figure 3b and supplemental Figure S3). The captured eluate was resolved on an SDS-PAGE stained gel. A very faint band migrating around 25 kDa was excised from the polyacrylamide gel and analyzed by mass spectrometry. A total of six proteins were identified from the 12C9 capture and six proteins from the MBP tag control capture (Table 1). The major component of the 12C9-captured sample was MAP_4145 with 24 peptides mapping to this protein, three of them unique (Table 1). BLAST analysis suggested the gene product is a membrane protein, however, PSORTb 3.0 analysis [22] could not predict a definitive location, even though two internal alpha helices were found. Nonetheless, our previous analysis with 12C9 on membrane- and cytosol-enriched *Map* extracts demonstrated that the protein is present primarily in membrane extracts [4].

**Figure 4:**
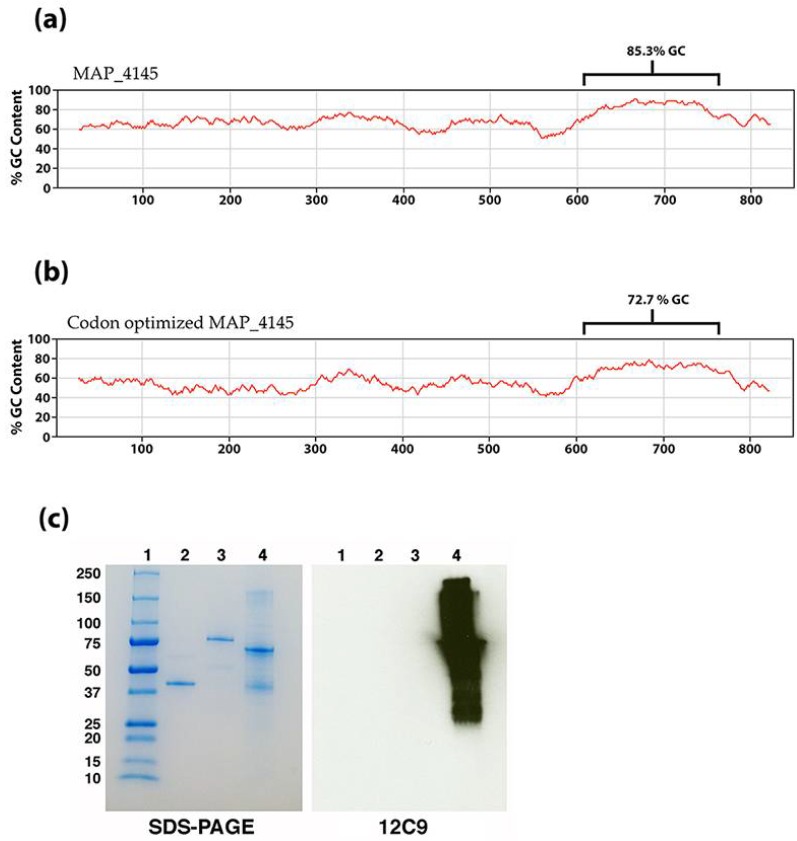
Codon optimized MAP_4145 reacts with mAb 12C9. The native (**a**) and codon optimized (**b**) percent G-C plots show the effect of replacing guanine or cytosine nucleotides with adenine and thymine at redundant locations. The brackets identify a 150 bp region that is especially rich in GC content. (**c**) SDS-PAGE and corresponding immunoblot shows that 12C9 reacts specifically and strongly to the codon optimized MAP_4145. Lanes: 1 = protein size markers, 2 = MAP_1609c, 3 = MAP_3842, and 4 = codon optimized MAP_4145.

To confirm the results of the immuno-capture and tandem mass spectrometry analysis, the gene must be cloned and expressed and tested with 12C9. However, this gene resisted amplification in all conditions tested due to the unusually high G + C content in the open reading frame. Specifically, there is a region of 150 base pairs starting at nucleotide 609 that has a G + C content of 85.3% (Figure 4a), which resulted in a melting temperature above 80 °C. To overcome this obstacle, the complete 849-bp gene was codon optimized, synthesized and cloned into an *E. coli* expression vector. The codon optimized MAP_4145 effectively lowered the G + C content of the 150-bp region from 85.3% to 72.7% (Figure 4b). The expressed protein was immunoblotted and confirmed that 12C9 does react to MAP_4145 (Figure 4c).

The binding targets for many of these *Map* mAbs have now been identified. This new information, combined with those from our earlier studies, are summarized in Table 2. This represents the most complete catalog of monoclonal antibodies to *Map* proteins available for research studies. With this new information, we can uniquely examine these proteins for relative abundance within the bacterial cell and determine their presence in defined fractions of *Map*.

### 3.5. Epitope Mapping of Selected mAbs to Map Proteins

**Figure 5:**
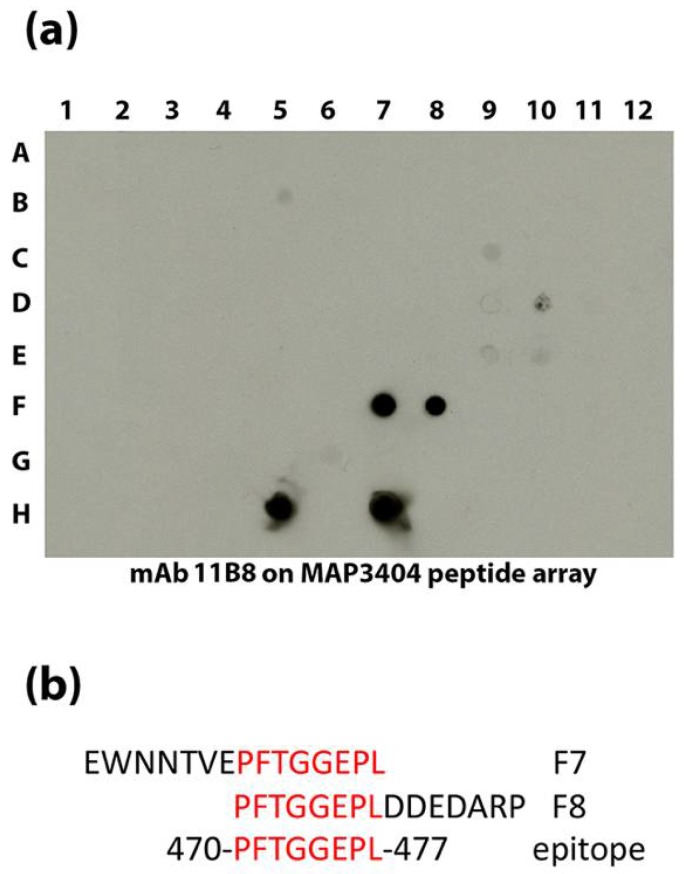
The epitope for 11B8 is defined to eight amino acids. An overlapping peptide library was spotted onto nitrocellulose and exposed to 11B8 (**a**). In addition to the native protein spotted in H5 and H7, the antibody reacts to overlapping peptides spotted in F7 and F8. The 15-amino acid peptides in F7 and F8 are shown (**b**) along with the overlapping amino acids highlighted in red. The consensus epitope is shown at the bottom and occurs at 470 to 477 in MAP_3404.

Multiple mAbs to MAP_3936 and MAP_3404 were obtained in this study. Therefore, the epitopes were identified to determine if single or multiple epitopes were recognized in each protein. Of the three mAbs to groEL2 encoded by MAP_3936, two react to epitopes near the center of the protein (10C12 and 11F8), while 7A6 binds between amino acid 408 and 541 at the C-terminal end based on reactivity to truncated fragments of the protein (supplemental Figure S4). An overlapping peptide array (supplemental Table S1) was used to identify epitopes to the five MAP_3404 mAbs. Using this array, the 11B8 epitope was defined to eight amino acids (Figure 5). The other four antibodies had complex reactivity patterns that were difficult to determine the precise epitopes, but the reactivity patterns were similar enough that it appears likely to be a similar epitope shared among them (supplemental Figure S5).

The 12C9 epitope was mapped to the center region of the MAP_4145 protein. Portions of the MAP_4145 were cloned such that the N-terminal, C-terminal, and center sections of the protein were independently expressed. Only the center 142 amino acids from 71 to 212 was detected by the antibody (supplemental Figure S6).

### 3.6. Monoclonal Antibody Use in Mycobacterial Proteomics

**Figure 6:**
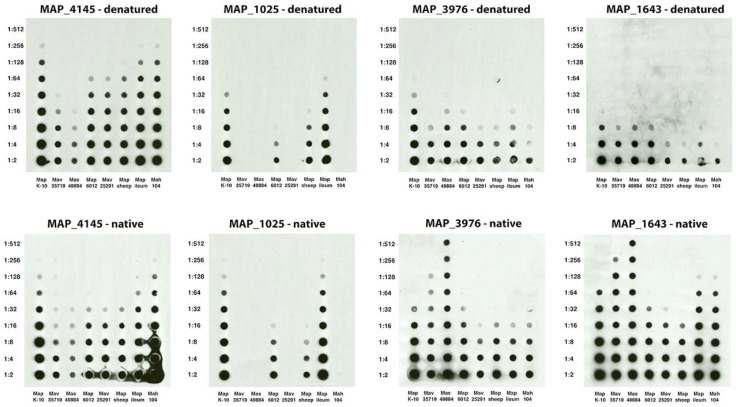
Relative abundance of select *Map* proteins in whole cell extracts of mycobacteria. Shown are a series of identically loaded dot blots with dilutions indicated in the left margin and MAC complex strain indicated beneath each blot. The protein detected and condition tested are shown above each blot. Note that relative abundance of each protein can be determined across each prep and between preps.

With the antigen identity known for many of the mAbs from this and other studies, they can now be used in many applications. One application is to quickly test the quality and relative abundance of selected proteins in fractionated proteomic preparations. As an example, several mycobacterial sonicated preparations were analyzed for relative abundance of protein in denaturing and non-denaturing conditions (Figure 6). There is a striking change in relative abundance of proteins from strain to strain in the two conditions. It is important to note that the lower abundance in the denatured condition for isocitrate lyase (MAP_1643) and lipoprotein anchoring transpeptidase (MAP_3976) is more likely due to heat susceptibility of the epitope rather than a change of abundance. The epitope to the proline rich antigen (MAP_1025) was less affected, but still showed a slight decrease in reactivity in the denatured condition. The opposite is observed for the MAP_4145 protein. There is a slight increase in the reactivity when the protein is denatured compared to native. The epitope in MAP_1025 is specific for only the paratuberculosis subspecies, even though the protein itself is present in other MAC complex strains [5], yet abundance similarities exist between the ileum *Map* isolate and the K-10 strain. Likewise, the levels are similar between the sheep strain and bovine isolate 6012. MAP_4145 and MAP_1025 protein levels are essentially identical in K-10 and present in higher relative abundance than is the transpeptidase (MAP_3976).

## 4. Discussion

Antibodies are powerful tools in diagnostic and molecular immunology studies. However, their utility is limited if their corresponding antigen is unknown. A recent application of *Map* monoclonal antibodies is for immunomagnetic separation; however, no effort was made to identify their binding partners [2]. In addition, mAbs that bind to lipoarabinomannan (LAM) in the mycobacterial cell wall were used to identify tuberculosis-infected patients using an electrochemiluminescence assay on urine samples [23]. The best capture and detection mAb pairs were identified from a collection of anti-LAM mAbs and used to greatly increase the sensitivity and specificity in a point-of-care test for tuberculosis. A similar capture mAb-detection mAb approach could be applied to Johne’s disease diagnosis using the mAbs described herein. In this study, we identified the corresponding antigens for all of the monoclonal antibodies described in our earlier study [4] as well as many from the current study. The complete list of *Map* monoclonal antibodies and their corresponding antigens is listed in Table 2.

While several of the mAbs were identified by screening a phage expression library, others could not be identified by this approach despite repeated efforts using two independent phage libraries. This suggests the biased nature of the library construction. If restriction sites used in library cloning do not result in in-frame expression clones then *E. coli* will not express the protein and thus the antigen cannot be identified. The protein array is an unbiased approach, but getting a complete array is not trivial. The antigen to 12C9 could not be identified despite using molecular approaches including protein array analysis and screening phage expression libraries, all of which had proven unsuccessful. Only through biochemical and immunological approaches that include affinity purification and immunoprecipitation was the 12C9 binding antigen identified.

It is well-known that *E. coli* does not tolerate heterologous membrane protein expression [24] and this might partially explain why more antigens were not identified by this screening method. Another possibility is that the phage library was not fully representative and thus did not contain every expressed gene. Besides underrepresentation, phage expression library approaches have a few pitfalls to be aware of. The expression library artificially forces expression of any cloned segment, regardless of whether or not the sequence codes for a real protein. Thus mimotopes, which mimic a real epitope, can be present in these libraries leading the researcher on a “wild goose chase”. In one such case, our group identified the one antibody (17A12) that is truly specific to *Map* and does not cross-react with other closely related *M. avium* subspecies [5]. However, when screening a phage expression library to identify the corresponding protein, a mimotope present in an unannotated region of the *Map* genome was obtained from four independent, positive library clones. Only after defining the mimotope down to seven amino acids were we able to use that information to screen the *Map* genome to identify the real epitope which had a one amino acid difference from the mimotope [5]. 

Another pitfall appears when the expression library was constructed for an organism that does not actually produce the epitope. This happened when a group was attempting to identify a protein associated with gastric cancer development [11]. They thought that the antigen was produced in a human gastric cell line (MGC0803) since mice that were immunized with MGC0803 yielded a mAb that would specifically react with tumor cells. However, the library screen failed and the subsequent immunocapture experiment identified the antigen which was actually produced by *Mycoplasma* [11]. In the current study, 11 mAbs all bound to annotated proteins in the expression library and could easily be confirmed by testing corresponding recombinant proteins. 

Likely the reason why MAP_4145 was never identified from either DNA expression library is because of the very high GC content in that gene. All attempts to amplify and clone MAP_4145 failed until it was codon optimized to lower the GC content. Once this was accomplished, the amplification, cloning, and expression in *E. coli* were easily obtained. Because of this difficulty in heterologous expression of MAP_4145, immunomagnetic precipitation was the only method to successfully capture and identify this protein.

Mapping of the fatty acid desaturase (MAP_2698c) epitope using an overlapping peptide array approach did not work. Furthermore, the native protein was not susceptible to heat denaturation while the full-length recombinant was susceptible. The reasons for this differential reactivity could be further defined, but our results strongly suggest this antibody recognizes a conformational epitope. 

With these mAb binding partners known, the locations of the antigens within the bacilli that were determined experimentally can now be compared with bioinformatic protein location predictions such as PSORTb analysis. The mAbs 12C9 and 14G11 both bound to antigens present in membrane-enriched fractions of *Map* [4] while their corresponding antigens MAP_4145 and MAP_3976 were predicted to be membrane located by PSORTb analysis [22] in this study (Table 2). For the desaturase (MAP_2698c) protein, PSORTb could not predict its location, however, experimental evidence clearly suggests this protein is in the membrane [4,25]. Conversely, 9G10 and 11G4 clearly bound to isocitrate lyase and DnaK, two proteins in the cytoplasm [4], and PSORTb correctly predicted that location as well. This demonstrates that PSORTb is an accurate predictor of protein location within bacteria.

Antibodies were used to determine abundance in whole cell extracts of mycobacteria belonging to the MAC complex. This can be readily applied to any fractionated protein preparation. Researchers can quickly determine presence or absence of specific proteins and examine how each protein’s abundance changes in different conditions and strains.

## Figures and Tables

**Table 1:** Mass spectrometry results.

Identified Proteins	Accession Number	Weight	Locus Tag	Total Read Counts		No. of Unique Peptides	
				MBP	12C9	MBP	12C9
Uncharacterized protein M. paratuberculosis (strain ATCC BAA-968/K-10) GN=MAP_4145 PE = 4 SV = 1	Q73SC9_MYCPA	30 kDa	MAP_4145	0	24		3
60 kDa chaperonin 2 M. paratuberculosis (strain ATCC BAA-968/K-10) GN=groL2 PE = 3 SV = 4	CH602_MYCPA	57 kDa	MAP_3936	10	0	3	
Uncharacterized oxidoreductase MAP_3007 M. paratuberculosis (strain ATCC BAA-968/K-10) GN=MAP_3007 PE = 3 SV = 1	Y3007_MYCPA	30 kDa	MAP_3007	12	0	4	
35kd_ag M. paratuberculosis (strain ATCC BAA-968/K-10) GN=MAP_2855c PE = 4 SV = 1	Q73W06_MYCPA	29 kDa	MAP_2855c	0	4		2
Uncharacterized protein M. paratuberculosis (strain ATCC BAA-968/K-10) GN=MAP_2720c PE = 4 SV = 1	Q73WE0_MYCPA	33 kDa	MAP_2720c	0	4		2
Haloalkane dehalogenase M. paratuberculosis (strain ATCC BAA-968/K-10) GN=dhmA PE = 3 SV = 1	DHMA_MYCPA	34 kDa	MAP_2057c	0	4		2
Wag31 M. paratuberculosis (strain ATCC BAA-968/K-10) GN=wag31 PE = 4 SV = 1	Q73YR6_MYCPA	28 kDa	MAP_1889c	6	0	2	
Malate dehydrogenase M. paratuberculosis (strain ATCC BAA-968/K-10) GN=mdh PE = 3 SV = 1	MDH_MYCPA	35 kDa	MAP_2541c	0	4		2
Uncharacterized protein M. paratuberculosis (strain ATCC BAA-968/K-10) GN=MAP_0398c PE = 4 SV = 1	Q744E3_MYCPA	25 kDa	MAP_0398c	0	4		2
Ornithine carbamoyltransferase M. paratuberculosis (strain ATCC BAA-968/K-10) GN=argF PE = 3 SV = 1	OTC_MYCPA	34 kDa	MAP_1365	6	0	3	
Pyrroline-5-carboxylate reductase M. paratuberculosis (strain ATCC BAA-968/K-10) GN=proC PE = 3 SV = 1	Q73ST2_MYCPA	30 kDa	MAP_3991	4	0	2	
Hsp90 co-chaperone Cdc37 OS=Bos taurus GN=CDC37 PE = 2 SV = 1	CDC37_BOVIN (+1)	45 kDa		4	0	2	

**Table 2:** Antibodies and corresponding antigen characteristics.

mAb	mAb	Cognate			Antigen	Antigen	Reference
Name	Isotype ^a^	Antigen	Epitope Location	Antigen Description	Size (kDa)	Location ^b^	
17A12-3B10 ^c^	IgG1	MAP_1025	28-HPGGQQP-34	Proline rich antigen (Pra); RDD family protein	25.0	Cytoplasmic membrane	Bannantine et al. [5]
14C5 ^d^	IgG2a	MAP_1272c	Between amino acids 26 and 74	NlpC/P60 domain protein; peptidoglycan hydrolase	29.2	Cytoplasmic membrane	Bannantine et al. [19]
8G6 ^d^	IgG1	MAP_1272c	Between amino acids 74 and 128	NlpC/P60 domain protein; peptidoglycan hydrolase	29.2	Cytoplasmic membrane	Bannantine et al. [19]
9G10	IgG2a	MAP_1643	C-terminal half of protein	isocitrate lyase, AceAb	85.2	Cytoplasmic	Bannantine et al. [4]
11F6	IgG1	MAP_1643	C-terminal half of protein	isocitrate lyase, AceAb	85.2	Cytoplasmic	Bannantine et al. [4]
8C6	ND	MAP_1643		isocitrate lyase, AceAb	85.2	Cytoplasmic	This study ^e^
10D2	IgG2b	MAP_1643		isocitrate lyase, AceAb	85.2	Cytoplasmic	This study
1C8	ND ^f^	MAP_1643		isocitrate lyase, AceAb	85.2	Cytoplasmic	This study
13E1-4E3 ^g^	IgG1	MAP_2121c	Binds only to full length protein	Major membrane protein (MMP)	33.6	Cytoplasmic membrane	Bannantine et al. [8]
8G2-2A10 ^g^	IgG1	MAP_2121c	Between amino acids 78 and 153	Major membrane protein (MMP)	33.6	Cytoplasmic membrane	Bannantine et al. [8]
14G3	IgG2a	Unknown			~40		Bannantine et al. [4]
14D4	IgG3	MAP_2698c	Conformational epitope ^f^	Fatty acid desaturase	31.5	Unknown ^h^	Bannantine et al. [4]
6C9	IgG1	MAP_3060c		Electron transfer protein, a-subunit	32.0	Cytoplasmic	This study
3G5	No react ^i^	MAP_3404		Acetyl-CoA carboxylase, biotin carboxylase subunit	64.3	Cytoplasmic	This study
7C8	IgG2a	MAP_3404		Acetyl-CoA carboxylase, biotin carboxylase subunit	64.3	Cytoplasmic	This study
9H3	IgG2b	MAP_3404	316-TEETAGIDLVLQQFK-330	Acetyl-CoA carboxylase, biotin carboxylase subunit	64.3	Cytoplasmic	This study
12E4	IgG1	MAP_3404		Acetyl-CoA carboxylase, biotin carboxylase subunit	64.3	Cytoplasmic	This study
11B8	IgG1	MAP_3404	470-PFTGGEPL-477	Acetyl-CoA carboxylase, biotin carboxylase subunit	64.3	Cytoplasmic	This study
11G4	IgG1	MAP_3840	N-terminal half of protein	DnaK chaperone; Heat shock protein	66.5	Cytoplasmic	Bannantine et al. [4]
13A4	IgG2b	MAP_3840	Binds only to full length protein	DnaK chaperone; Heat shock protein	66.5	Cytoplasmic	Bannantine et al. [4]
7A6	IgG2a	MAP_3936		Molecular chaperone GroEL2	56.6	Cytoplasmic	This study
11F8	IgG2b	MAP_3936		Molecular chaperone GroEL2	56.6	Cytoplasmic	This study
10C12	IgG1	MAP_3936		Molecular chaperone GroEL2	56.6	Cytoplasmic	This study
14G11	IgG1	MAP_3976	Not determined	Lipoprotein anchoring transpeptidase	47.0	Cytoplasmic membrane	Bannantine et al. [4]
12C9	IgG1	MAP_4145	Between amino acids 71 and 212	Membrane protein with a short C-terminal domain	30.0	Cytoplasmic membrane	Bannantine et al. [4]
4B6	IgG1	Unknown	Unknown	A highly conserved, unidentified mycobacterial protein	~42	Cytoplasmic	Bannantine et al. [4]
12F9	IgG1	Unknown					This study
5E6	IgG1	Unknown					This study
10D4	IgG1	Unknown					This study
11F9	IgG1	Unknown					This study
13G7	ND ^f^	Unknown					This study
2D12	IgG1	Unknown					This study
1F2	IgG2a	Unknown					This study
4A9	IgG1	Unknown					This study
4G1	IgM	Unknown					This study
11F5	ND	Unknown		Binds to a protein of similar size to MAP_3404	~65		This study

^a^ Only the heavy chain isotype is indicated. All antibodies listed here have the kappa light chain. ^b^ Antigen location was based on either PSORTb 3.0 results or immunoblot results against membrane- and cytoplasmic-enriched protein preps, or both. ^c^ mAb 17A12 was obtained using a membrane enriched extract as the immunizing antigen. ^d^ mAbs 14C5, 8G6 were obtained using recombinant MAP_1272c as the immunizing antigen. ^e^ This study means the antibody has not been published prior to this article. ^f^ No reactivity observed on a peptide array of MAP2698c except the full-length protein, suggesting a conformational epitope. ^g^ mAbs 13E1-4E3 and 8G2-2A10 were obtained using recombinant MAP_2121c at the immunizing antigen. ^h^ The PSORTb 3.0 prediction for MAP_2698c is unknown; however, the literature suggests it is in the membrane (He and DeBuck 2012) ^i^ No react = No reaction to isotyping antibodies. Was repeated three times.

## Supplementary Materials

The following are available online at http://www.mdpi.com/2076-2607/6/4/127/s1, Figure S1: mAbs 11F8, 7A6 and 10C12 react to the MAP_3936 coding sequence within phage clone #4-2a, Figure S2: mAb 6C9 binds to MAP_3060c, Figure S3: The antigen that binds 12C9 was captured in a specific manner, Figure S4: Location of antibody epitopes to the MAP_3936 groEL2 protein, Figure S5: MAP_3404 epitope mapping using an overlapping peptide array, Figure S6: Location of the 12C9 antibody epitope to the center region of MAP_4145, Table S1: Recombinant protein assignments on the dot blot array shown in Figure 2B, Table S2: Peptide library sequences and spot/well locations for MAP_2698c and MAP_3404, Table S3: Monoclonal antibody reactivity to mycobacterial species.
