# Supplementary material for: Membrane and Cytoplasmic Proteins of Mycobacterium avium subspecies paratuberculosis that Bind to Novel Monoclonal Antibodies
Source: Microorganisms. 2018 Dec 11;6(4):127. doi: 10.3390/microorganisms6040127 (PMC6313528; doi:10.3390/microorganisms6040127)
Supplement: Supplementary file 1 [file microorganisms-06-00127-s001.zip › Supplemental figures.pdf]

(a)

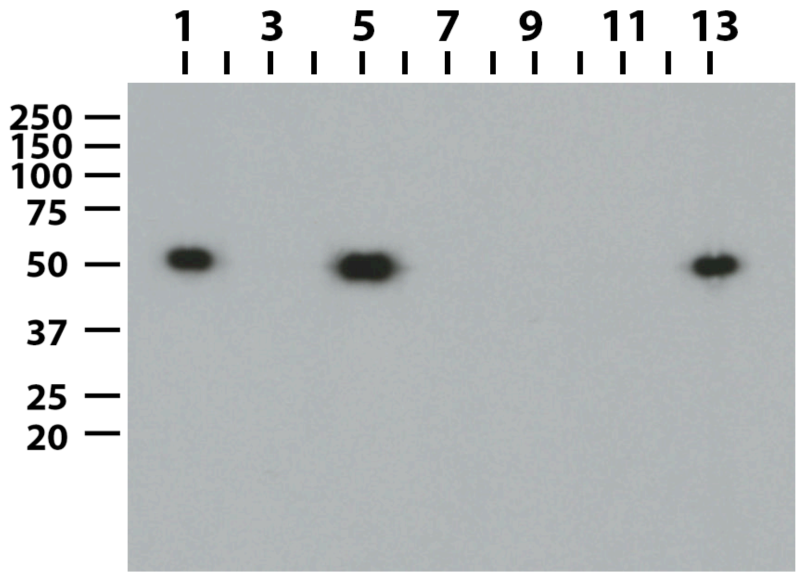

(b)

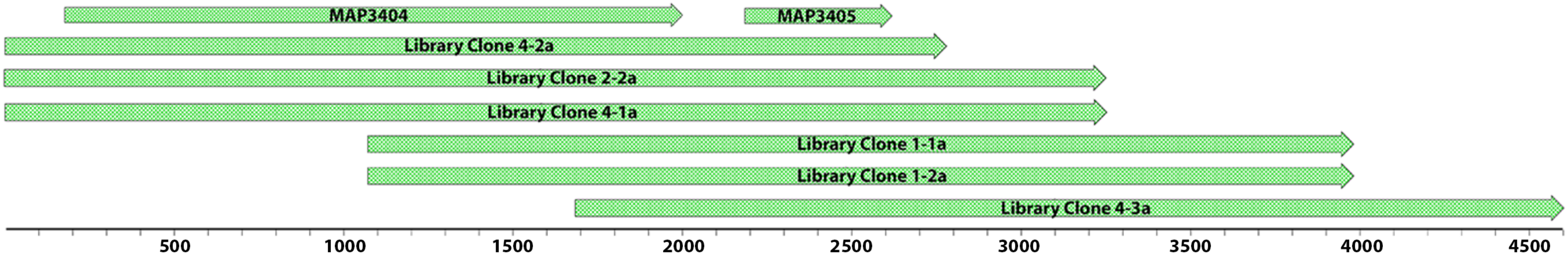

Supplemental Figure S1. mAbs 11F8, 7A6 and 10C12 react to the MAP_3936 coding sequence within phage clone #6a. Shown is a preparatory immunoblot containing IPTG induced phage clone #6a exposed to seven mAbs used in the library screen loaded in independent slots of a slot blotting device (**a**). Kilodalton size markers are shown in the left margin. Slot assignments: 1=11F8, 3=3G5, 5=7A6. 7=11B8, 9=9H3, 11=12E4, 13=10C12. These antibodies were mapped to defined sections of the protein (Figure S4). In a separate experiment, the inserts of 6 overlapping clones are aligned in (**b**) along with the MAP_3404 coding sequence that reacted with five mAbs in this study. No mAbs reacted with MAP_3405. Clone sequences are represented by green arrows which show the direction of lacZ promoter transcription within the plasmid clone and the length relative to a base pair scale.

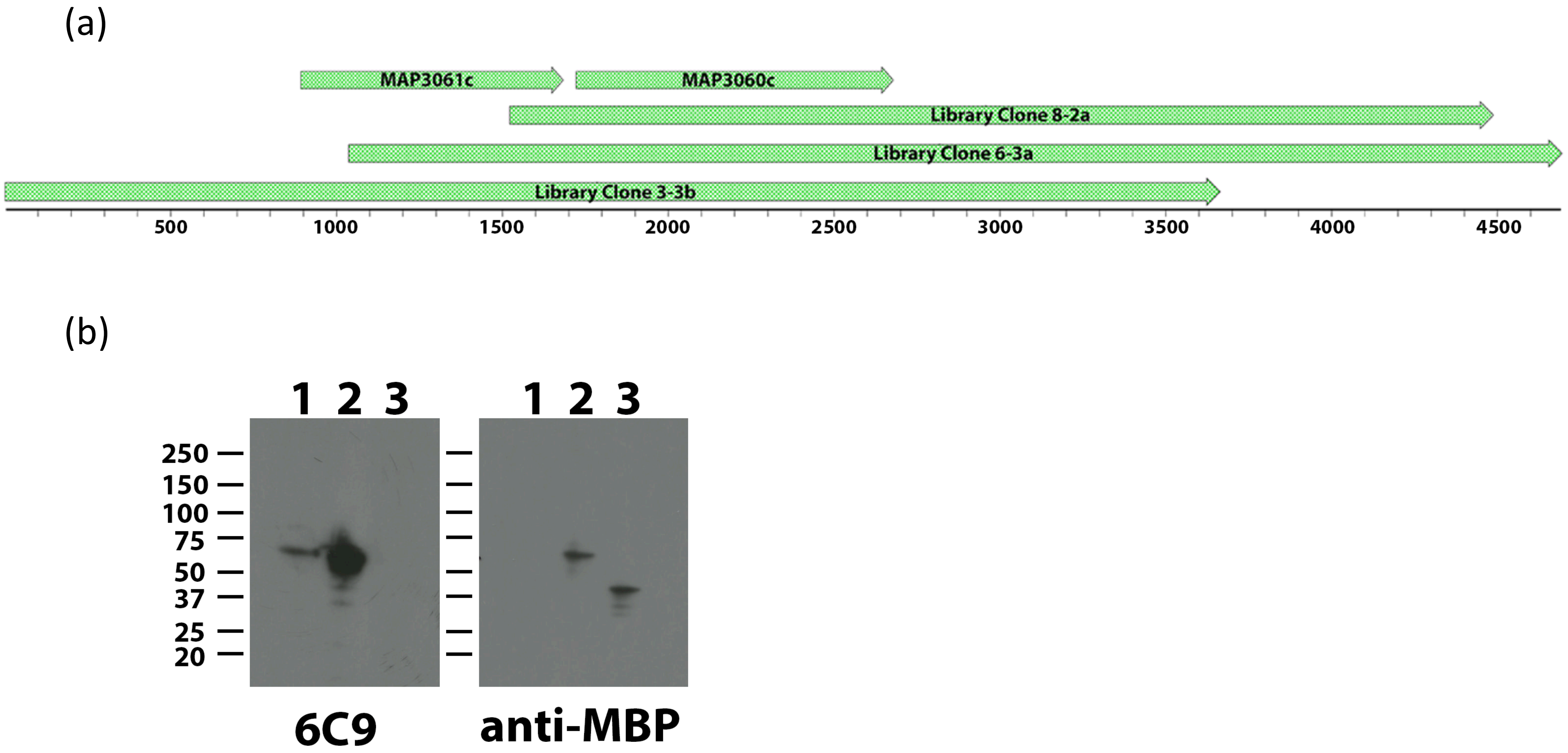

Supplemental Figure S2. mAb 6C9 binds to MAP_3060c. Plaques that reacted to 6C9 were purified and subcloned into pBluescript-SK for sequencing. Clone sequences are represented by green arrows which show the direction of lacZ promoter transcription within the plasmid clone and the length relative to a base pair scale (**a**). MAP_3061c and MAP3060c are the annotated genes within the library clone inserts. An alignment of three overlapping clones relative to the two coding sequences MAP_3061c and MAP_3060c is shown. mAb 6C9 was found to react to the MAP_3060c gene product by immunoblot (**b**). Lane assignments for (**b**) are: uninduced (lane 1) and IPTG induced *E. coli* (MAP3060c) (lane 2), MBP-LacZ control (lane 3).

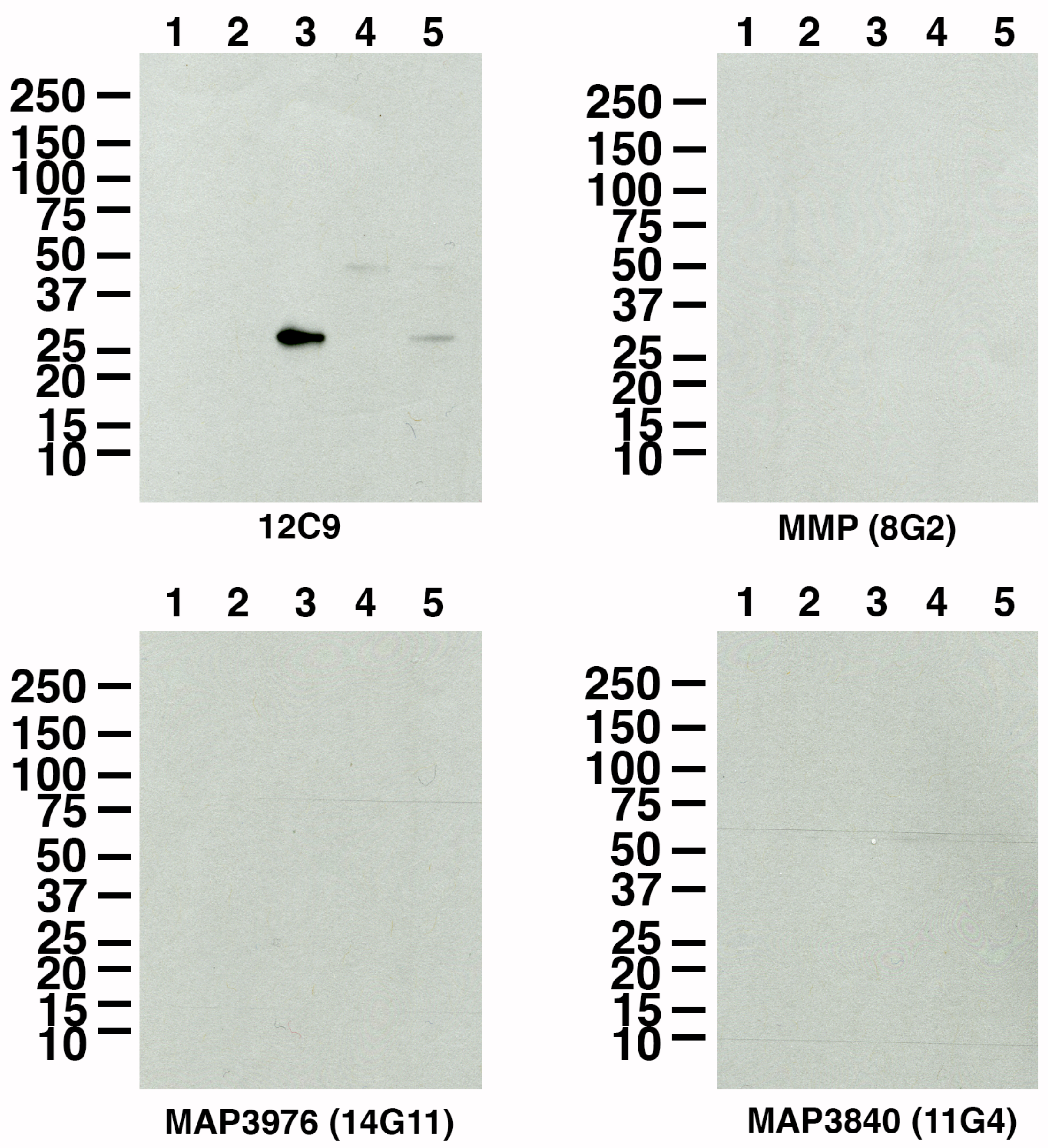

Supplemental Figure S3. The antigen that binds 12C9 was captured in a specific manner. Shown are four identical immunoblots probed with monoclonal antibodies indicated. The captured antigen only reacts with the 12C9 antibody. Also note that more antigen is present from the direct method (lane 3 of the 12C9 blot) when compared to the indirect method (lane 5). Lane assignments for all immunoblots: 1=Protein size markers, 2=combined Dynabead washes (direct method), 3=SDS boiled beads (direct method), 4=combined Dynabead washes (indirect method), 5=SDS boiled beads (indirect method).

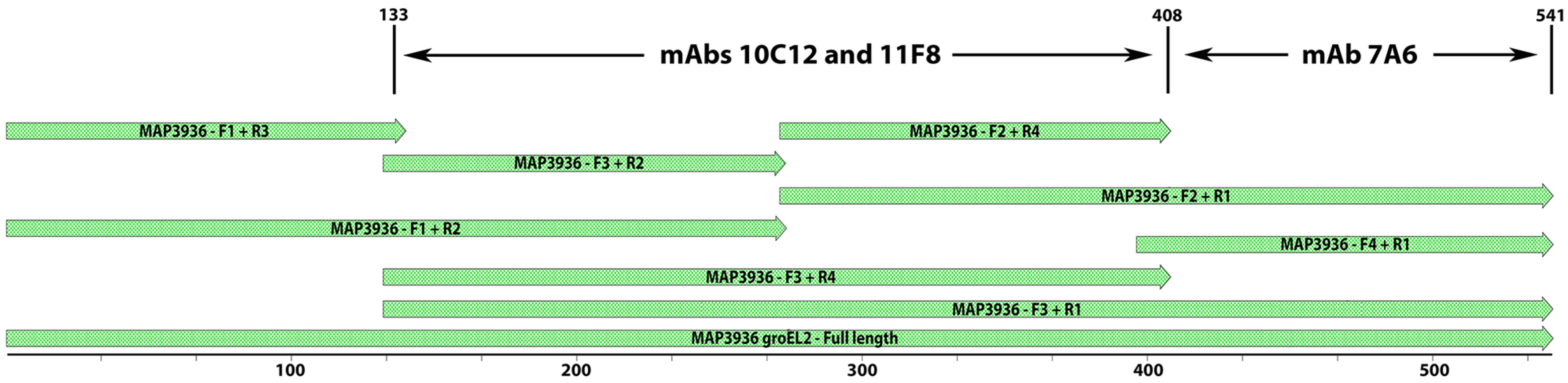

Supplemental Figure S4. Location of antibody epitopes to the MAP_3936 groEL2 protein. The full length gene along with 8 truncated segments were cloned and expressed in *E. coli* as shown schematically to scale. Sizes of the truncated expression products and relative positions within the full length protein are depicted on a scale showing amino acid number. Purified recombinant peptides representing defined sections of the protein were immunoblotted and probed with the 3 mAbs indicated. Reactivity patterns associated with each mAb suggest that mAbs 10C12 and 11F8 bind to an epitope located in the center of the protein between amino acids 133 and 408, whereas 7A6 binds to an epitope on the C-terminal end between amino acids 408 and 541.

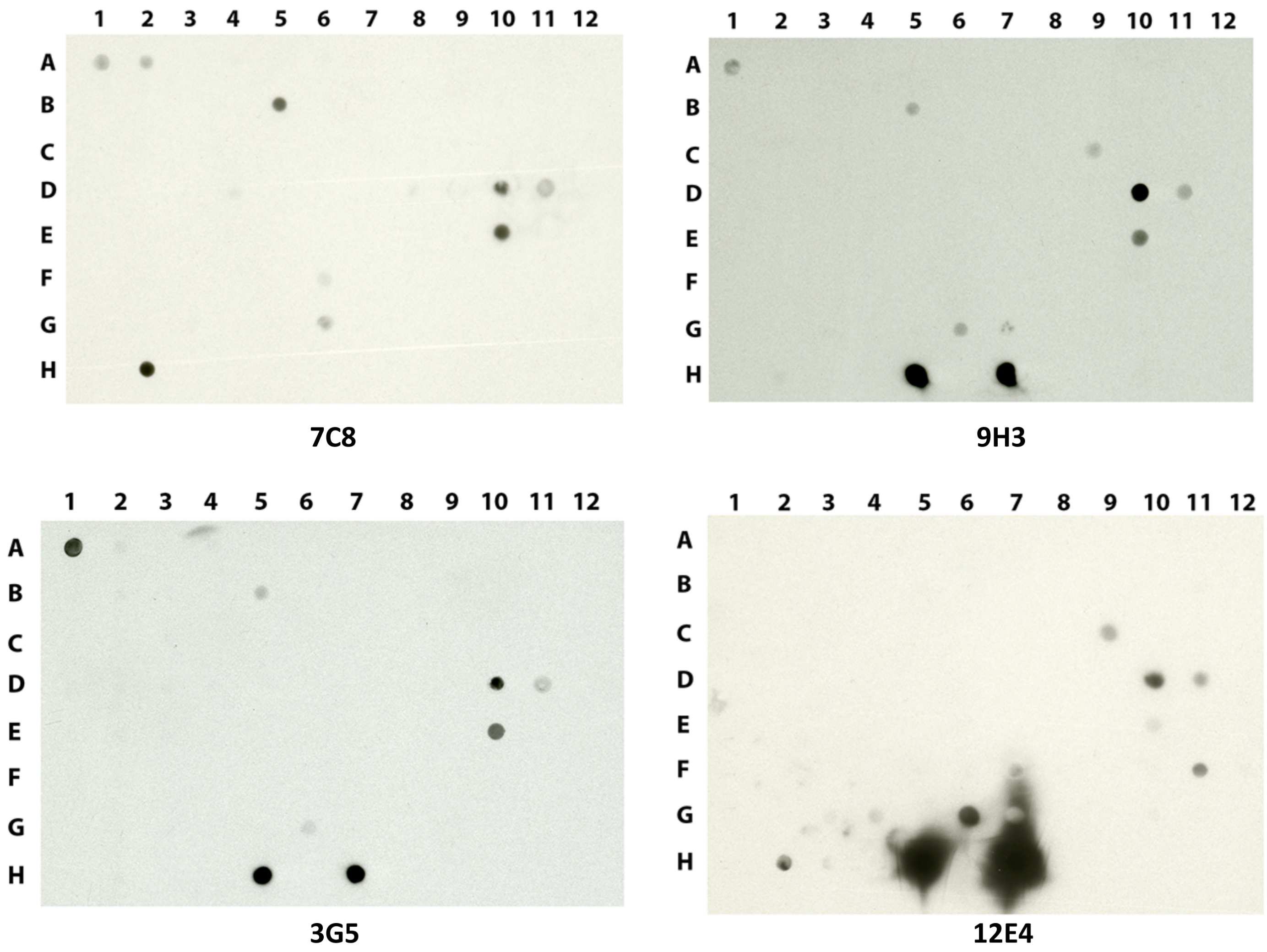

Supplemental Figure S5. MAP_3404 epitope mapping using an overlapping peptide array. Shown are four arrays exposed to the mAbs indicated beneath each array. The K-10 extract served as the positive control and was loaded in H5 and H7 for all arrays except 7C8. The reactive peptides common to all four include D10, D11, E10, and G6. The peptides in D10 and D11 overlap with each other.

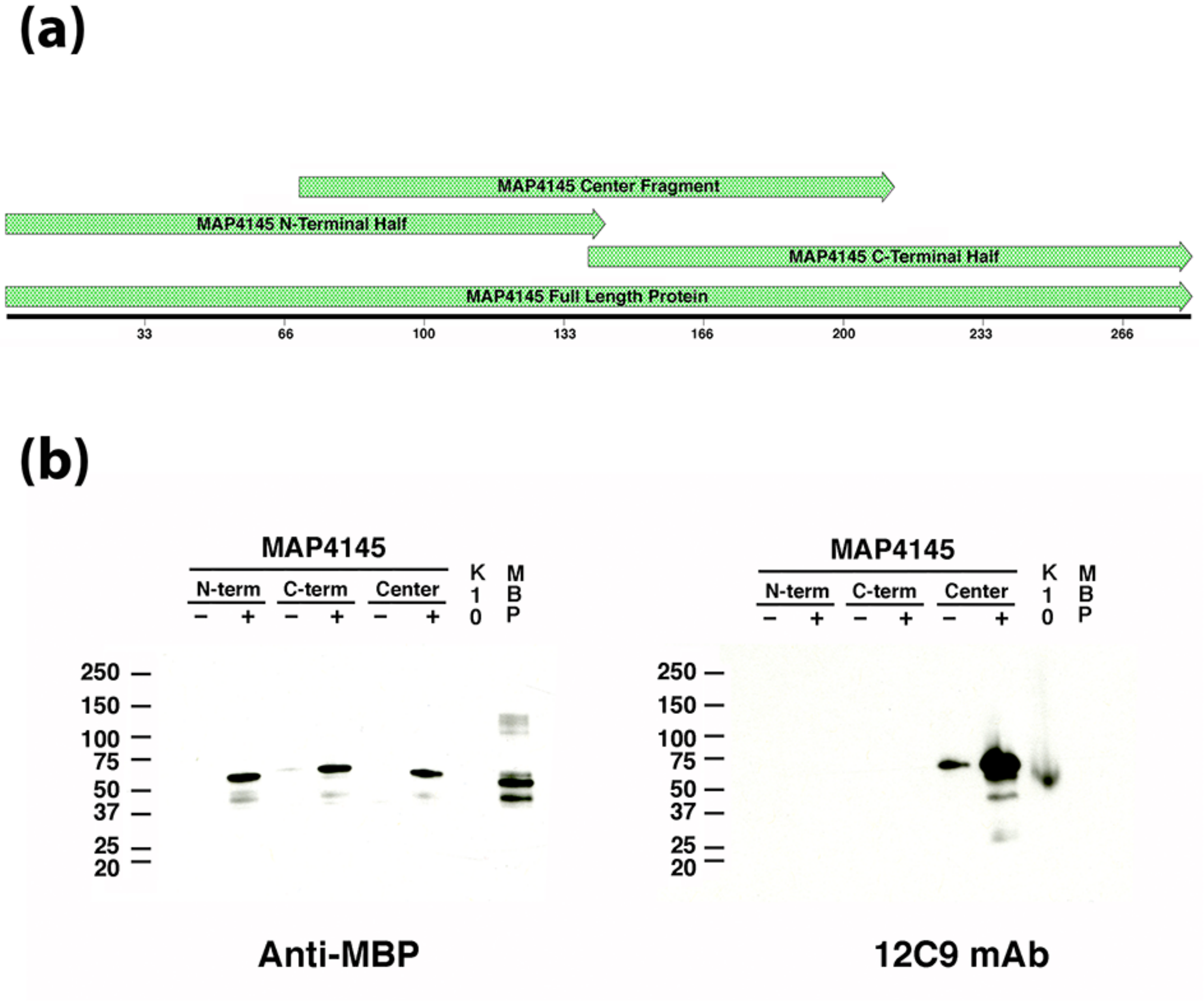

Supplemental Figure S6. Location of the 12C9 antibody epitope to the center region of MAP_4145. Three clones were produced and expressed in *E. coli* as shown schematically to scale in (a). The truncated protein sizes and relative positions within the full length protein are depicted on a scale showing amino acid number. Purified recombinant peptides representing the N-terminal half, central region, and C-terminal half of the protein were immunoblotted and probed with a mAb to the affinity tag (MBP) and 12C9 as indicated beneath each blot. The blot probed with anti-MBP (left blot) shows the recombinant proteins are only expressed under IPTG-induced conditions (+) and not uninduced (-). The MBP-LacZ control protein is present in the far right lane. The positions of the standards are indicated in the left margin. The K-10 extract lane is labelled K10. Note that only the center fragment is detected by the 12C9 antibody along with the native protein in K-10.
